# Fully automated measurement on coronal alignment of lower limbs using deep convolutional neural networks on radiographic images

**DOI:** 10.1186/s12891-022-05818-4

**Published:** 2022-09-17

**Authors:** Xianghong Meng, Zhi Wang, Xinlong Ma, Xiaoming Liu, Hong Ji, Jie-zhi Cheng, Pei Dong

**Affiliations:** 1 Department of Radiology, Tianjin Hospital, Jiefangnan Road, Hexi District, Tianjin, 300211 China; 2 Department of Orthopaedics, Tianjin Hospital, Jiefangnan Road, Hexi District, Tianjin, 300211 China; 3 Beijing United Imaging Research Institute of Intelligent Imaging, Haidian District, Yongteng North Road, Beijing, 100089 China; 4 United Imaging Intelligence (Beijing) Co., Ltd, Haidian District, Yongteng North Road, Beijing, 100089 China; 5 Shanghai United Imaging Intelligence Co., Ltd, Xuhui District, YunJin Road, Shanghai, 200232 China

**Keywords:** Lower limbs, Full-length X-ray, Alignment measurement, Deep convolutional neural networks

## Abstract

**Background:**

A deep convolutional neural network (DCNN) system is proposed to measure the lower limb parameters of the mechanical lateral distal femur angle (mLDFA), medial proximal tibial angle (MPTA), lateral distal tibial angle (LDTA), joint line convergence angle (JLCA), and mechanical axis of the lower limbs.

**Methods:**

Standing X-rays of 1000 patients’ lower limbs were examined for the DCNN and assigned to training, validation, and test sets. A coarse-to-fine network was employed to locate 20 key landmarks on both limbs that first recognised the regions of hip, knee, and ankle, and subsequently outputted the key points in each sub-region from a full-length X-ray. Finally, information from these key landmark locations was used to calculate the above five parameters.

**Results:**

The DCNN system showed high consistency (intraclass correlation coefficient > 0.91) for all five lower limb parameters. Additionally, the mean absolute error (MAE) and root mean squared error (RMSE) of all angle predictions were lower than 3° for both the left and right limbs. The MAE of the mechanical axis of the lower limbs was 1.124 mm and 1.416 mm and the RMSE was 1.032 mm and 1.321 mm, for the right and left limbs, respectively. The measurement time of the DCNN system was 1.8 ± 1.3 s, which was significantly shorter than that of experienced radiologists (616.8 ± 48.2 s, *t* = -180.4, *P* < 0.001).

**Conclusions:**

The proposed DCNN system can automatically measure mLDFA, MPTA, LDTA, JLCA, and the mechanical axis of the lower limbs, thus helping physicians manage lower limb alignment accurately and efficiently.

**Supplementary Information:**

The online version contains supplementary material available at 10.1186/s12891-022-05818-4.

## Introduction

Knee osteoarthritis, commonly afflicting middle-aged and elderly women, is caused by many factors, such as heredity and obesity [1]. There is a high incidence of lower limb malalignment in patients that suffer osteotomy around the knee joint, unicompartmental replacement, and total knee arthroplasty [2, 3]. Additionally, osteotomy, lengthening, or both can also be performed on patients with unequal lengths of both lower limbs, mal-union and non-union fractures of the lower limbs, and on patients with chronic osteomyelitis and bone tumours. Therefore, preoperative measurements of lower limb alignment in the standing position are critical since they may directly influence surgical treatment options.

The most measured parameters in the lower limbs include mechanical lateral distal femur angle (mLDFA), medial proximal tibia angle (MPTA), lateral distal tibia angle (LDTA), joint line convergence angle (JLCA), and lower limbs mechanical axis [4]. At present, doctors measure these lines and angles on X-ray films manually for both lower limbs in the standing position. However, the measurement typically takes 10–15 min and has low accuracy and repeatability [5]. With the updating of software and hardware, digital X-rays are being increasingly used to project the lower limbs. After scanning, the image is transmitted to the picture archiving and communication system for measurements, and the repeatability and reliability of the measurement are improved [6]. Currently, some commercial software programmes can measure these angles with higher stability, but they have some shortcomings, such as high cost and inconsistent measurement results [7].

Artificial intelligence (AI) is widely used in medical image processing and analysis; furthermore, it has been deeply explored and has applications in the musculoskeletal system, such as for disease triage, parameter optimisation, image segmentation, measurements, diagnosis, and prognosis [8–10]. Bier et al. [11] proposed a deep convolutional neural network (DCNN) algorithm to detect multiple anatomical landmarks in pelvic X-ray images from arbitrary viewing directions. Galbusera et al. [12] presented a fully convolutional neural network (CNN) algorithm to characterise an additional differentiable spatial-to-numerical layer to measure the parameters of T4-T12 kyphosis, L1-L5 lordosis, Cobb angle, pelvic incidence, sacral slope, and pelvic tilt.

Therefore, we present a two-stage AI model to automatically locate 20 landmarks in full-length X-ray films of the lower limbs. Considering these key points, we calculate and measure several lower limb parameters, including mLDFA, MPTA, LDTA, JLCA, and mechanical axis of the lower limbs. The contributions of our study are as follows:

1) The proposed DCNN model can provide reliable and reproducible measurements of lower limb parameters; 2) The proposed model can significantly shorten then measurement time.

## Materials and methods

All procedures in this study involving human participants were performed in accordance with the ethical standards of the Institutional Review Board and the 1964 Helsinki Declaration and its later amendments or comparable ethical standards. Approval from the Institutional Review Board of our hospital was obtained, with the project approval number of “2021 Medical Review 088”. The HIPAA requirements were followed. Informed consent was not required.

### Subjects

**Fig. 1:**
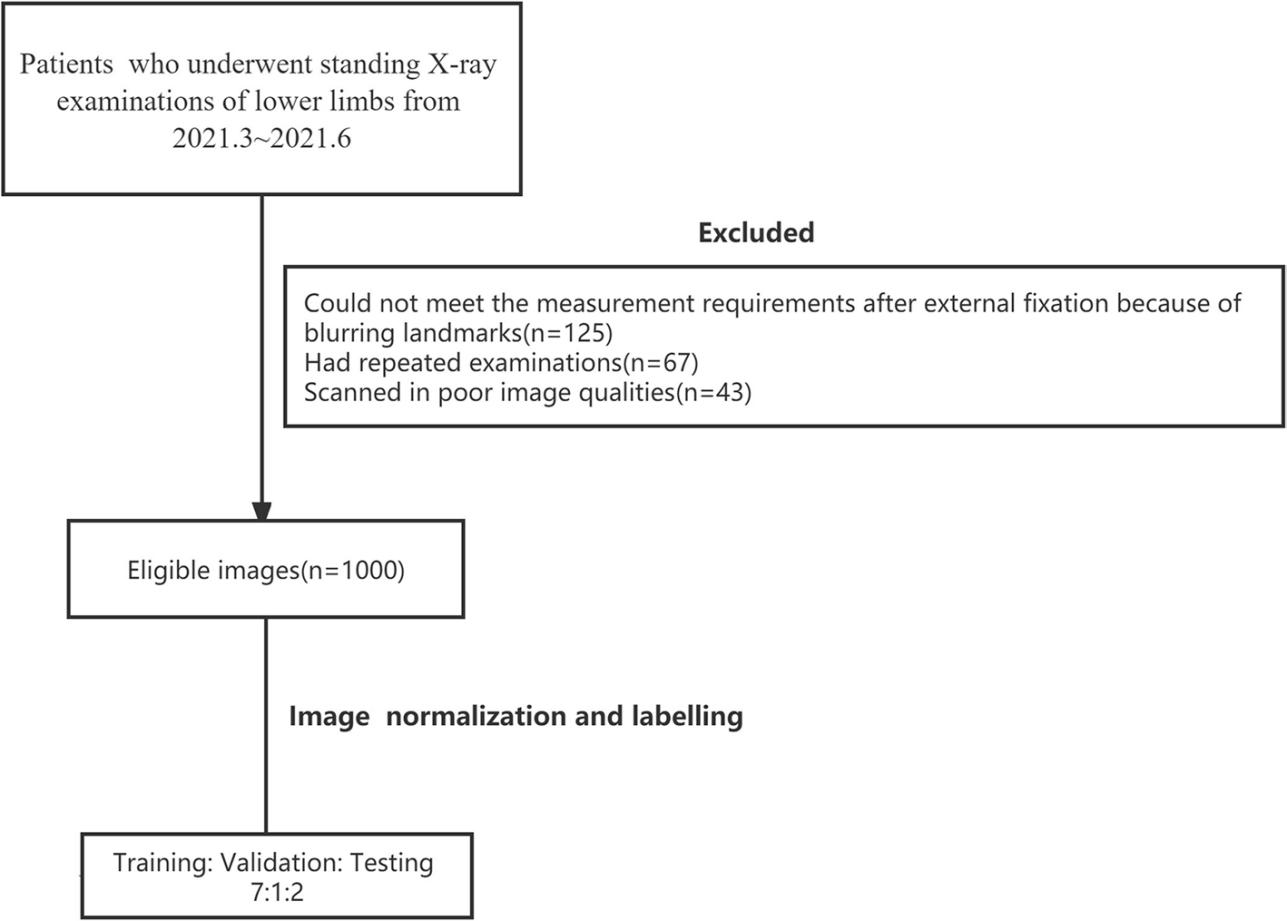
Flowchart of patient inclusion and exclusion

In this study, we selected patients who underwent standing X-ray examinations of the lower limbs from the period of March 2021 to June 2021. The exclusion criteria were as follows: (1) patients who could not meet the measurement requirements after external fixation because of blurring landmarks, (2) patients who had repeated examinations, and (3) patients who had poor image quality. A total of 1000 patients were enrolled in this study (Fig. 1).

### X-ray examinations and ground truth labelling

A double-planar X-ray scanner (Discovery XR656, GE Healthcare, Milwaukee, WI, USA) was used to perform full-length X-ray examinations of the lower limbs in the standing position. The patients stood naturally: both hands held both sides of a shelf, kept their feet shoulder-width apart, the median sagittal plane of the body was perpendicular to the detector and the horizontal plane, the coronal plane of the body was perpendicular to the horizontal plane, the patella was facing forward, and the lower limbs were completely extended. Tube voltage, tube current, and target-film distance were set to 75 kV, 25 mAs, and 180 cm, respectively. The height of included X-rays ranged from 4411 to 6430 (median = 5638.5, standard deviation = 334.4). The width of the included X-rays ranged from 1200 to 2020 (median = 2004, standard deviation = 54.9), and the pixel spacing ranged from 0.1851 mm to 0.1891 mm (median = 0.1884 mm, standard deviation = 0.002 mm).

The measurement parameters included: 1) the mechanical axis of the femur, the line between the centre of the femoral head and the lowest point of the intercondylar fossa of the femur, where the centre of the femoral head is determined by Mose concentric circles, 2) the mechanical axis of the tibia, i.e. the line between the midpoint of the intercondylar ridge of the tibia and the midpoint of the talus, 3) mLDFA: lateral angle of the tangent line of the distal femoral articular surface intersects the mechanical axis of the femur, 4) MPTA: medial angle of intersection of articular surface tangent of tibiae plateau with mechanical axis of tibia, 5) LDTA: lateral angle of intersection of tangent of distal articular surface of tibia and mechanical axis of tibia, 6) JLCA: angle of intersection of tangent lines of the distal femur and tibial plateau, and 7) mechanical axis of the lower limb, i.e. the distance between the centre of the femoral head and midpoint of the talus. The ground truth of the training, validation, and test sets was measured by two experienced radiologists (YQL, experience in imaging diagnosis of musculoskeletal system for 10 years, and ZFL, experience in imaging diagnosis of musculoskeletal system for 7 years). We considered the average values from the two radiologists as the ground truth to decrease the individual differences between markers [13]. Finally, another senior radiologist (XHM, engaged in imaging diagnosis of musculoskeletal system for 12 years) reviewed all the generated ground truth and revised some inconsistent cases.

All measurements were performed independently on a local measuring tool based on Python 3.6. The radiologist first opened the software and imported full-length X-ray images of both lower limbs. Next, the centre of the femoral head, the lowest points of the lateral and medial condyles of the femur, the lowest point of the intercondylar fossa of the femur, the lateral and medial point of the tibial plateau, the midpoint of the intercondylar spine of the tibia, and the lowest points of the lateral and medial articular surface of the distal tibia were marked. To expand the application scope of the DCNN, patients who had total knee arthroplasty (TKA) were also enrolled in the study. The total number of the TKA cases was 234 patients in the entire dataset. We proportionally divided these TKA cases into training (*n* = 163), validation (*n* = 23), and test (*n* = 48) sets. For the TKA cases, we marked the medial and lateral points and the middle points between the medial and lateral points of the joint prosthesis in the distal femur and proximal tibia. Following marking, the software automatically calculated and displayed the mLDFA, MPTA, LDTA, JLCA, and the mechanical axis of the lower limbs. Finally, the measured data were saved and exported to Excel.

### Data splitting, pre-processing, and augmentation

According to examination dates, we divided 70% of the enrolled X-rays as the training set (*n* = 700), 10% as the validation set (*n* = 100), and the remaining 20% of the X-rays were treated as the test set (*n* = 200).

To eliminate the scanning differences between subjects, we applied a series of pre-processing to normalise all the enrolled X-rays. First, we resampled the pixel spacing to 1 × 1 mm. Min–max normalisation was next used to scale pixel values. We also employed rotation as an augmentation strategy to increase the variance of the training set. In the training phase, the input X-rays were rotated at a random angle in the range of $-$ 5° to 5°.

### Deep learning methods of landmark location

**Fig. 2:**
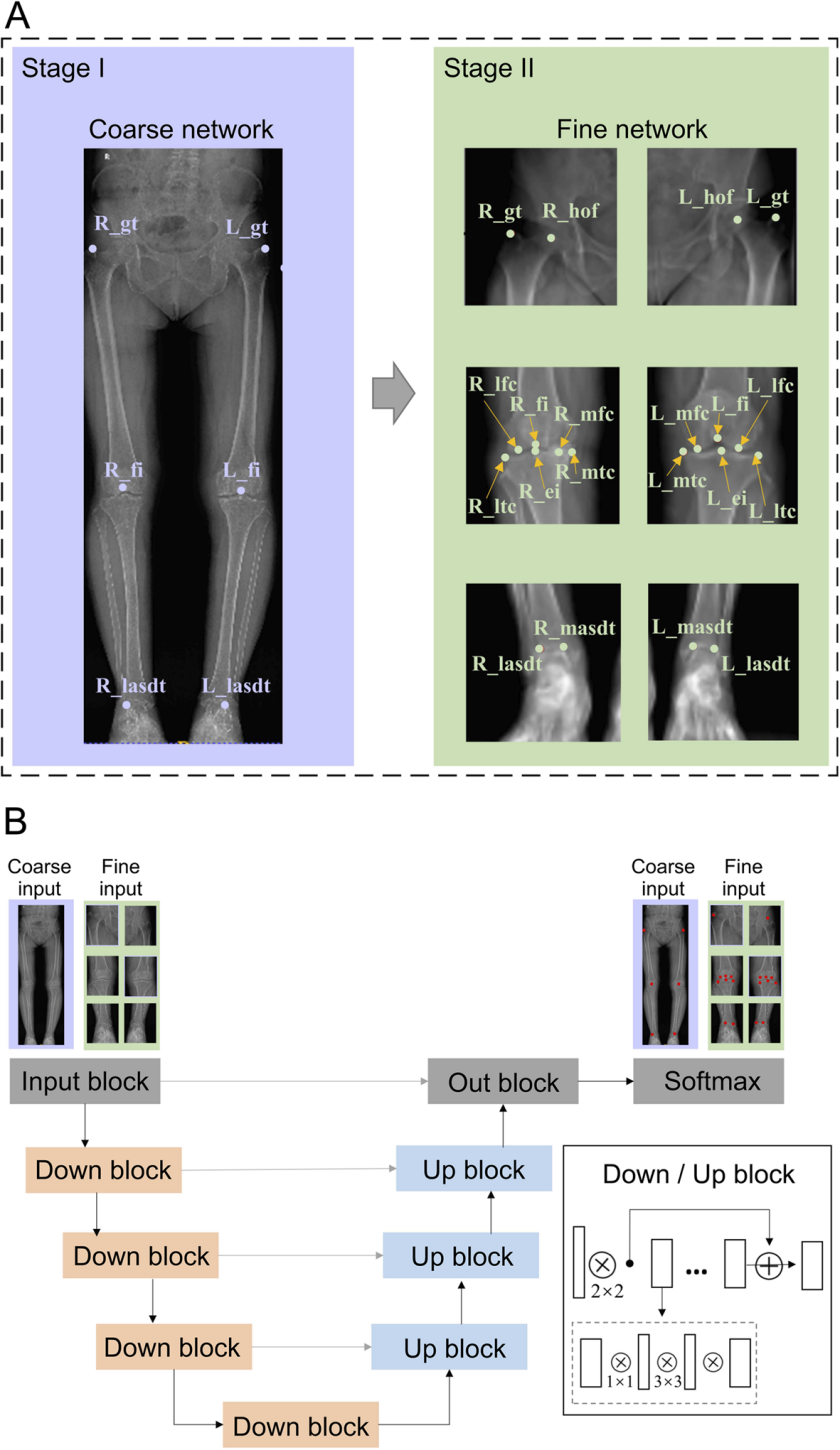
Architecture of VB-Net. R: right; L: left. hof: head of femur; gt: greater trochanter; lfc: lateral femoral condyle; mfc: medial femoral condyle; fi: fossa intercondyle; ltc: lateral tibial condyle; mtc: medial tibial condyle; ei: eminentia intercondyle; lasdt: lateral articular surface of the distal tibia; masdt: medial articular surface of the distal tibia

In this study, we employed the VB-Net [14] architecture as the basic model to build a coarse-to-fine system. The architecture of VB-Net is shown in Fig. 2. In the novel VB-Net, a bottle-neck structure replaces the conventional convolutional layers in the convolutional U-Net and thus contributes to a significant decrease in the model size. In this study, we considered a 20 × 20 region around each ground truth as the network input. In addition, we output the centre of the largest connected component in probability maps. In this manner, we realised the landmark detection using a segmentation network.

As shown in Fig. 2(a), the inputs for the coarse network were the full-length X-rays in a resolution of 1 × 1 mm. VB-Net first located the greater trochanter, intercondylar fossa of the femur, and the lateral malleolus on both the left and right sides of the X-ray, respectively. The size of the predicted region was 20 × 20 pixels. Then, to enable the fine networks to realise more accurate landmark location, we resampled the X-rays to 0.25 × 0.25 mm. Three pairs of image patches were extracted around the above-mentioned regions. The cropped field of view (FOV) was 180 mm^2^ for the greater trochanter regions, 128 mm^2^ for the intercondylar fossa regions, and 104 mm^2^ for the lateral malleolus regions. The fine VB-Nets were constructed to precisely locate the greater trochanter, head centre of the femur in greater trochanter-centric patches, lateral femoral condyle, intercondylar fossa, medial femoral condyle, lateral tibial condyle, eminentia intercondylaris, medial tibial condyle in intercondylar fossa-centric patches, lateral malleolus, and medial malleolus in lateral malleolus-centric patches. The segmented FOV of the fine networks was 5 mm^2^ (20 × 20 pixels).

In this study, we used the training set to build a two-stage network. We set the loss function as the focal loss and the constant learning rate as 0.0001 based on the validation set. We used the test set to evaluate the performance of the proposed AI-aided lower limb measuring system. The deep learning algorithm was developed on PyTorch with an NVIDIA GeForce GTX TITAN X graphic card.

Automatic measurement of lower limb alignment.

**Fig. 3:**
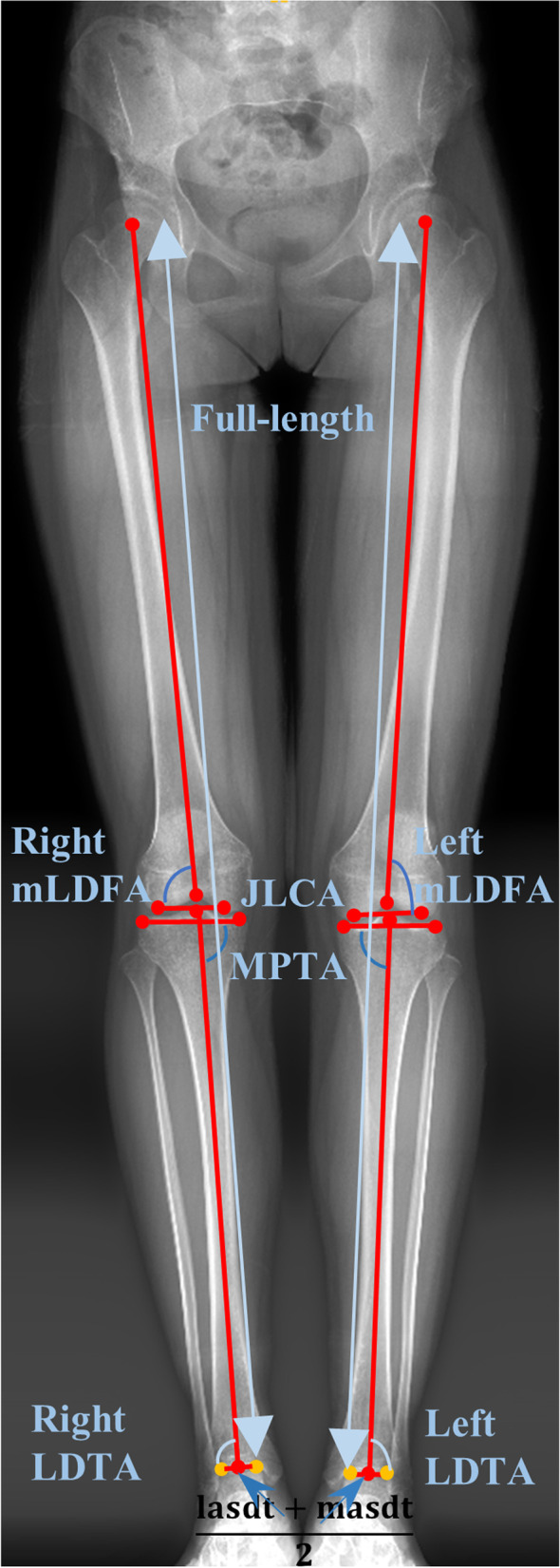
Definition of lower limb alignment parameters. mLDFA: mechanical lateral distal femur angle; MPTA: medial proximal tibia angle; LDTA: lateral distal tibia angle, JLCA: joint line convergence angle; lasdt: lateral articular surface of the distal tibia; masdt: medial articular surface of the distal tibia

In this study, we aimed to obtain mLDFA, MPTA, LDTA, JLCA, and the mechanical axis of the lower limbs for preoperative measurements. The related parameters of lower limb alignment can be calculated automatically using the 10-pairs core regions. The calculations are detailed in Fig. 3.

### Statistical analysis

Statistical analyses were performed using SPSS 26.0 software (version 26.0; SPSS Inc., Chicago, IL, USA). For key landmark location estimation, we determined the percentage of points of the correct key (PCK) [15] with a threshold of 3 mm. Furthermore, some metrics were employed to estimate the angle prediction performance of the DCNN system and human experts. Intraclass correlation coefficients (ICCs) and Pearson correlation were used to analyse the correlation between the measurements of the DCNN system and the ground truth. For the variability analysis, the mean absolute error (MAE) and root mean squared error (RMSE) were calculated. The measurement time of the DCNN system and the ground truth were compared using a paired-samples *t*-test with 95% confidence interval (CI). Statistical significance was set at *P* < 0.05. Additionally, to visually demonstrate the distribution of the metrics, Bland–Altman plots were drawn.

## Results

### Results of key-point location

**Fig. 4:**
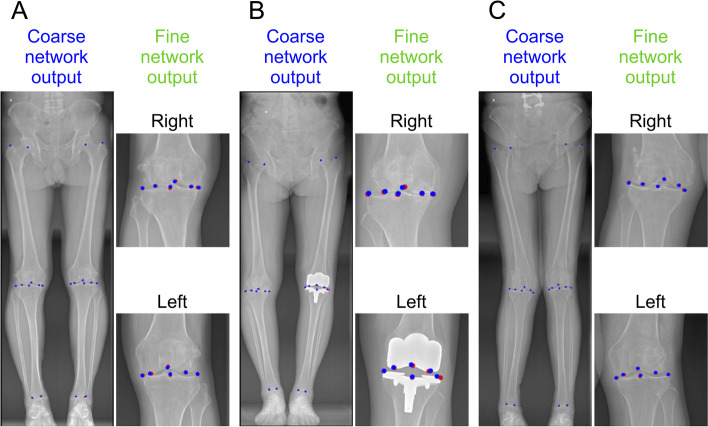
Key points location results of the proposed AI model. Red dot: ground truth; Blue dot: DCNN system

**Fig. 5:**
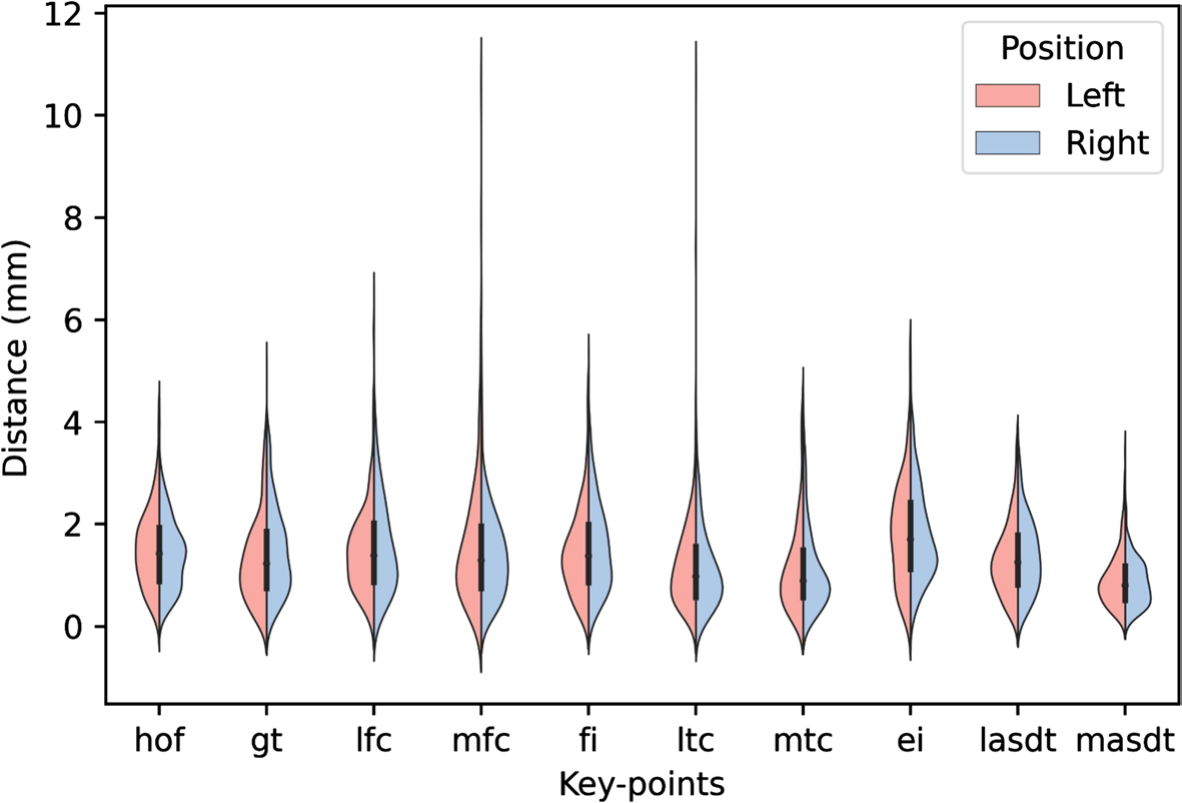
Violin plot of DCNN system and ground truth. hof: head of femur; gt: greater trochanter; lfc: lateral femoral condyle; mfc: medial femoral condyle; fi: fossa intercondyle; ltc: lateral tibial condyle; mtc: medial tibial condyle; ei: eminentia intercondyle; lasdt: lateral articular surface of the distal tibia; masdt: medial articular surface of the distal tibia

We evaluated the key landmark location performance of the proposed coarse-to-fine networks on the test set. The results are shown in Fig. 4, where the red dots represent the radiologists’ annotations, and the blue dots represent the DCNN systems’ predictions. We used PCK to explore the correct percentage of all the detected points. If the absolute distance between the radiologists’ annotations and AI predictions was less than 3 mm, we regarded the detected point as a desirable output. Table 1 shows the PCK of our AI model. From Table 1, we can discover that PCK for all the landmarks exceeded 90%. The detailed error distributions between AI predicted key points and the ground truth for 20 points are shown as a violin plot in Fig. 5.

**Table 1:** PCK of DCNN system

**Key points**	**head of femur**		**greater trochanter**		**lateral femoral condyle**		**medial femoral condyle**		**intercondylar fossa**	
Position	Left	Right	Left	Right	Left	Right	Left	Right	Left	Right
PCK	96.5%	98.9%	94.5%	93.0%	95.9%	90.9%	90.9%	96.5%	94.9%	92.9%
**Key** **points**	**lateral tibial condyle**		**medial tibial condyle**		**eminentia intercondyly**		**lateral malleolus**		**medial malleolus**	
Position	Left	Right	Left	Right	Left	Right	Left	Right	Left	Right
PCK	97.4%	90.9%	90.9%	94.9%	90.0%	88.9%	96.%	97.9%	99.5%	100%

*PCK* Points of correct key

### Results of lower limb measurements

**Fig. 6:**
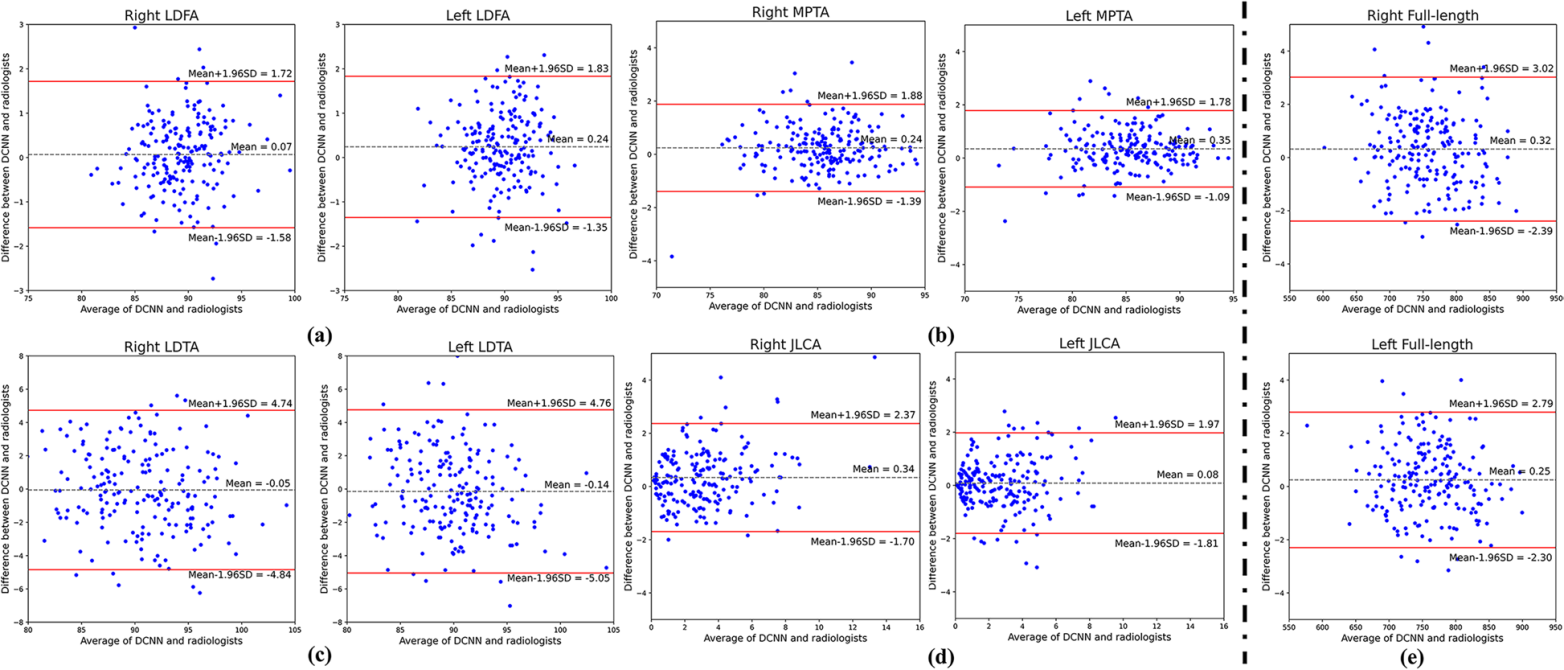
Bland–Altman plots of angles and mechanical axis of lower limb measurements

Following the key-point detection, the above-mentioned parameters were automatically calculated. A detailed comparison is presented in Table 2. For all the measurements (four angles and the whole length of the lower limbs), the *P*-values of the Mann–Whitney U Test showed no significant differences between AI calculations and the ground truth. ICCs and Pearson correlation also demonstrated a strong correlation between the model and radiologists. MAEs for the four angles were all smaller than 2.1°. The MAEs for the whole length of the lower limbs were 1.124 mm and 1.032 mm for the right side and the left side, respectively. The Bland–Altman plots of the angles and mechanical axis of the lower limb measurements are shown in Fig. 6.

**Table 2:** The results of parameter calculation of lower limbs

		***P* value** **(95% CI)**	**ICC**	**Pearson correlation**	**MAE**	**RMSE**
**mLDFA ( °)**	Right	0.212 (0.180, 0.225)	0.982	0.964	0.642	0.849
	Left	0.194 (0.163, 0.202)	0.980	0.961	0.656	0.847
**MPTA ( °)**	Right	0.312 (0.193, 0.327)	0.988	0.976	0.638	0.872
	Left	0.332 (0.209, 0.344)	0.991	0.982	0.602	0.813
**LDTA ( °)**	Right	0.312 (0.193, 0.327)	0.939	0.887	1.986	2.451
	Left	0.394 (0.209, 0.344)	0.915	0.850	2.048	2.512
**JLCA ( °)**	Right	0.194 (0.158, 0.212)	0.949	0.907	0.825	1.092
	Left	0.224 (0.207, 0.261)	0.942	0.891	0.716	0.969
**mechanical axis of lower limbs (mm)**	Right	0.234 (0.207, 0.261)	1.000	1.000	1.124	1.416
	Left	0.194 (0.167, 0.237)	1.000	1.000	1.032	1.321

*mLDFA*, Mechanical lateral angle of distal femur *MPTA*, Medial angle of proximal tibia *LDTA*, Lateral angle of distal tibia *JLCA*, Joint convergent angle of articular surface *ICC*, Intraclass correlation coefficient *MAE*, Mean absolute error *RMSE*, Root mean squared error

### Results of the measurement time

The measurement time of the DCNN system was 1.8 ± 1.3 s, which was significantly shorter than that of the ground truth (616.8 ± 48.2 s, *t* = -180.9, *P* < 0.001).

In summary, the proposed coarse-to-fine DCNN can significantly enhance the measuring efficiency with a comparable accuracy for multi-measurements in lower limbs.

## Discussion

In this study, we used a coarse-to-fine DCNN system to realise ten pairs of key landmark locations in full-length X-ray films for the lower limbs. The study demonstrated that the DCNN model can automatically and accurately measure mLDFA, MPTA, LDTA, JLCA, and mechanical axis of the lower limbs. The presented method can provide reliable and repeatable measurements and significantly shorten the measurement time.

Researchers have increasingly paid attention to semi-automatic and automatic measurements on X-ray films. Some authors have utilised commercial software to measure the angles and lengths of plain films. Schröter et al. [16] found that the two digital planning software programmes, mediCAD® and PreOPlan®, showed high interrater reliability in deformity analysis and digital planning of osteotomies of the knee joint, and experience of the observer had no influence on the results. Segev et al. [17] found that digital measurements with the TraumaCad® system are reliable in terms of intra- and inter-observer variability, making it a useful method for the analysis of pathology on radiographs in paediatric orthopaedics, including pelvic, lower limb, and spine deformities. Sled et al. [7] used a Horizon Surveyor custom software programme to semi-automatically measure frontal plane lower limb alignment, demonstrating that alignment measurements using a bone landmark-based approach and a computer programme were highly reliable among multiple readers. However, these commercial software packages have many drawbacks. First, they are expensive, making them difficult to popularise. Additionally, they require radiologists or clinicians to manually mark the measurement key points on the image, which is time consuming. Finally, the measurement errors among these software packages are large and cannot meet clinical demands [5, 6].

Therefore, an increasing number of studies have used DCNNs to automatically measure these angles and lengths on plain films. Schock et al. [18] used a DCNN algorithm to measure hip-knee-ankle angle and femoral anatomic-mechanical angle automatically and quantitatively and the measurements were as precise and accurate as manual reference measurements with 3–7 s. Recently, Simon et al. [19] tested the LAMA software, which was trained on over 15,000 radiographs from multiple centres using DCNN to measure the hip-knee-ankle angle, anatomical mechanical angle, JLCA, mLDFA, LDTA, mechanical lateral proximal femoral angle, MPTA, mechanical-axis-deviation, leg length, femur length, and tibia length. The software achieved an overall accuracy of 89.2% when comparing the AI outputs to those that were manually measured. AI vs. observers revealed a mean absolute deviation between 0.39° and 2.19° for angles and 1.45–5.00 mm for lengths. The ICC between AI and observers showed good reliability in all lengths and angles (ICC ≥ 0.87). In this study, PCK was used to evaluate the performance of landmark location. Table 1 demonstrates that the located accuracies are almost 90% for 10 pair candidates with threshold of 3 mm. For overall assessment, additional PCK with thresholds of 2.5 mm, 2 mm, 1.5 mm, and 1 mm are shown in Tables S1, S2, S3 and S4, respectively.

Based on the landmark location, five measurements of the lower limbs were calculated. Table S1 shows that there was no significant difference between manual annotation and DCNN system calculation. Figure S1 shows a patient with malalignment of left knee with genu varum. The distances between AI predictions and ground truth for left lateral femoral condyle, medial femoral condyle, intercondylar fossa, lateral tibial condyle, medial tibial condyle, and eminentia intercondyle were 2.56 mm, 2.98 mm, 2.16 mm, 1.47 mm, 1.21 mm, and 2.81 mm, respectively. The absolute errors between proposed method and ground truth for mLDFA, MPTA, LDTA, JLCA, and the mechanical axis of the lower limbs on the left side were 1.14°, 0.86°, 1.98°, 2.54°, and 0.04 mm. The above metrics illustrate that pathological changes may decrease the landmark location accuracy and further influence relative measurements. Therefore, including more extreme deformity cases is expected to enhance the robustness of the DCNN.

The ICCs between the ground truth and the DCNN predictions indicate a high consistency. Specifically, the MAE of the predicted mLDFA was 0.642° for the right limbs and 0.656° for the left limbs, and the errors were much lower, similar to a previous report. Nguyen et al. [20] reported that the MAE for mLDFA was 0.899° for the right limbs and 1.137° for the left limbs. In our study, the predicted MAE for MPTA of the right and left limbs was 0.638° and 0.602°, respectively, which are also better than the ones reported by Nguyen et al. [20] (the MAE for MPTA of right and left limbs was 1.146° and 1.032°, respectively). Additionally, Zheng et al. [21] determined the mechanical axis of the lower limbs using the AI model with an MAE of 4.5 mm, and our corresponding MAE values for the right and left legs were 1.124 mm and 1.032 mm, respectively. To further explore the different measuring performance, Tables S5 and S6 display the measurements for preoperative X-ray and TKA patients, respectively. The results in Tables S5 and S6 and the predicted landmarks of right leg in Figure S1 demonstrate that our model can show equivalent ability in lower limbs alignment for knee prosthesis inserting X-rays.

Some authors have also developed DCNN algorithms to measure other indices on plain films. Ye et al. [22] developed a deep learning-based system for automatic patellar height measurements using knee radiographs, which can predict the Insall–Salvati, Caton–Deschamps, modified Caton–Deschamps, and Keerati indexes automatically with high accuracy. Li et al. [23] used a mask regional CNN model to detect four key points that delineate the Sharp’s angle. Python-based utility software was applied to automatically draw and calculate the Sharp’s angle. The AI model can automatically measure the Sharp’s angle with a performance similar to that of orthopaedic surgeons but requires considerably less time.

This study has some limitations. First, the data of the study were derived from a single hospital; we did not use multi-centre measurement data, so the generalisation of the DCNN model needs to be confirmed in the future. Second, the sample size was relatively small, and the DCNN model may perform insufficiently; thus, we must expand the sample size, especially with data of patients with severe deformities of the lower limbs.

## Conclusions

In this paper, we proposed a DCNN system to automatically provide multiple measurements (mLDFA, MPTA, LDTA, JLCA, and length of mechanical axis) for lower limb alignment in X-rays. The MAEs of the four angles were all less than 2.1°. The MAE for the entire length of the lower limbs was approximately 1 mm. The total measured time was 1.8 ± 1.3 s. The results demonstrated that our method is reliable and repeatable with a significant increase in efficiency.

In the future, we will focus on the validation of the proposed lower limb alignment method in more medical centres and enhance the robustness of the DCNN system.

## Supplementary Information

**Additional file 1:** **Table S1.** PCK of DCNN system with threshold of 2.5 mm. **Table S2.** PCK of DCNN system with threshold of 2 mm. **Table S3.** PCK of DCNN system with threshold of 1.5 mm. **Table S4.** PCK of DCNN system with threshold of 1 mm. **Table S5.** Results of parameter calculation of preoperative lower limbs in the test set.** Table S6. **Results of parameter calculation of TKA lower limbs in the test set.** Fig S1.** The measurement differences between AI predictions and ground truth in a patient with genu varum of the left knee. The figure shows the differences between AI predictions and ground truth for left lateral femoral condyle, medial femoral condyle, fossa intercondyle, lateral tibial condyle, medial tibial condyle and eminentia intercondyle in this patient. Red dot: ground truth; Blue dot: DCNN system.

## Data Availability

The datasets used and/or analysed during the current study are available from the corresponding author on reasonable request.

## Abbreviations

AI: Artificial intelligence
CNN: Convolutional neural network
DCNN: Deep convolutional neural network
mLDFA: Mechanical lateral angle of distal femur
MPTA: Medial angle of proximal tibia
LDTA: Lateral angle of distal tibia
JLCA: Joint convergent angle of articular surface
FOV: Field of view
PCK: Points of correct key
ICC: Intraclass correlation coefficient
MAE: Mean absolute error
RMSE: Root mean squared error
