# Supplementary material for: Fully automated measurement on coronal alignment of lower limbs using deep convolutional neural networks on radiographic images
Source: BMC Musculoskelet Disord. 2022 Sep 17;23:869. doi: 10.1186/s12891-022-05818-4 (PMC9482267; doi:10.1186/s12891-022-05818-4)
Supplement: Supplementary file 1 — Additional file 1: Table S1. PCK of DCNN system with threshold of 2.5 mm. Table S2. PCK of DCNN system with threshold of 2 mm. Table S3. PCK of DCNN system with threshold of 1.5 mm. Table S4. PCK of DCNN system with threshold of 1 mm. Table S5. Results of parameter calculation of preoperative lower limbs in the test set. Table S6. Results of parameter calculation of TKA lower limbs in the test set. Fig S1. The measurement differences between AI predictions and ground truth in a patient with genu varum of the left knee. The figure shows the differences between AI predictions and ground truth for left lateral femoral condyle, medial femoral condyle, fossa intercondyle, lateral tibial condyle, medial tibial condyle and eminentia intercondyle in this patient. Red dot: ground truth; Blue dot: DCNN system. [file 12891_2022_5818_MOESM1_ESM.docx]

**Table S1. PCK of DCNN system with threshold of 2.5 mm**

| **Key points** | **head of femur** | | **greater trochanter** | | **lateral femoral condyle** | | **medial femoral condyle** | | **intercondylar fossa** | |
| --- | --- | --- | --- | --- | --- | --- | --- | --- | --- | --- |
| Position | Left | Right | Left | Right | Left | Right | Left | Right | Left | Right |
| PCK | 90.4% | 93.4% | 89.9% | 85.9% | 89.9% | 82.3% | 83.8% | 90.4% | 90.9% | 85.4% |
| **Key**  **points** | **lateral tibial condyle** | | **medial tibial condyle** | | **eminentia intercondyly** | | **lateral malleolus** | | **medial malleolus** | |
| Position | Left | Right | Left | Right | Left | Right | Left | Right | Left | Right |
| PCK | 94.9% | 92.9% | 91.4% | 91.9% | 74.7% | 78.8% | 93.9% | 93.9% | 98.0% | 100% |

**Table S2. PCK of DCNN system with threshold of 2 mm**

| **Key points** | **head of femur** | | **greater trochanter** | | **lateral femoral condyle** | | **medial femoral condyle** | | **intercondylar fossa** | |
| --- | --- | --- | --- | --- | --- | --- | --- | --- | --- | --- |
| Position | Left | Right | Left | Right | Left | Right | Left | Right | Left | Right |
| PCK | 76.3% | 79.8% | 83.8% | 75.8% | 78.8% | 70.2% | 74.7% | 78.3% | 79.3% | 70.7% |
| **Key**  **points** | **lateral tibial condyle** | | **medial tibial condyle** | | **eminentia intercondyly** | | **lateral malleolus** | | **medial malleolus** | |
| Position | Left | Right | Left | Right | Left | Right | Left | Right | Left | Right |
| PCK | 85.9% | 86.4% | 82.8% | 86.9% | 59.6% | 65.7% | 84.3% | 80.8% | 93.9% | 97.8% |

**Table S3. PCK of DCNN system with threshold of 1.5 mm**

| **Key points** | **head of femur** | | **greater trochanter** | | **lateral femoral condyle** | | **medial femoral condyle** | | **intercondylar fossa** | |
| --- | --- | --- | --- | --- | --- | --- | --- | --- | --- | --- |
| Position | Left | Right | Left | Right | Left | Right | Left | Right | Left | Right |
| PCK | 50.5% | 56.6% | 65.7% | 53.5% | 54.5% | 54.0% | 59.6% | 59.6% | 58.6% | 51.5% |
| **Key**  **points** | **lateral tibial condyle** | | **medial tibial condyle** | | **eminentia intercondyly** | | **lateral malleolus** | | **medial malleolus** | |
| Position | Left | Right | Left | Right | Left | Right | Left | Right | Left | Right |
| PCK | 71.7% | 75.8% | 74.2% | 76.3% | 39.4% | 47.0% | 67.7% | 58.6% | 87.4% | 92.4% |

**Table S4. PCK of DCNN system with threshold of 1 mm**

| **Key points** | **head of femur** | | **greater trochanter** | | **lateral femoral condyle** | | **medial femoral condyle** | | **intercondylar fossa** | |
| --- | --- | --- | --- | --- | --- | --- | --- | --- | --- | --- |
| Position | Left | Right | Left | Right | Left | Right | Left | Right | Left | Right |
| PCK | 26.8% | 31.8% | 42.9% | 34.3% | 32.8% | 32.8% | 37.4% | 35.4% | 30.8% | 31.8% |
| **Key**  **points** | **lateral tibial condyle** | | **medial tibial condyle** | | **eminentia intercondyly** | | **lateral malleolus** | | **medial malleolus** | |
| Position | Left | Right | Left | Right | Left | Right | Left | Right | Left | Right |
| PCK | 47.0% | 55.6% | 54.0% | 60.6% | 26.8% | 17.2% | 35.9% | 35.4% | 65.7% | 65.7% |

**Table S5. Results of parameter calculation of**

**preoperative lower limbs in the test set**

|  | | ***P* value** | **ICC** | **Pearson correlation** | **MAE** | **RMSE** |
| --- | --- | --- | --- | --- | --- | --- |
| **mLDFA ( °)** | Right | 0.172 | 0.961 | 0.961 | 0.644 | 0.819 |
|  | Left | 0.101 | 0954 | 0.958 | 0.687 | 0.882 |
| **MPTA ( °)** | Right | 0.081 | 0.974 | 0.976 | 0.595 | 0.769 |
|  | Left | 0.310 | 0.966 | 0.973 | 0.653 | 0.863 |
| **LDTA ( °)** | Right | 0.837 | 0.873 | 0.873 | 1.996 | 2.461 |
|  | Left | 0.435 | 0.844 | 0.849 | 2.036 | 2.413 |
| **JLCA ( °)** | Right | 0.301 | 0.896 | 0.903 | 0.822 | 1.001 |
|  | Left | 0.741 | 0.861 | 0.862 | 0.771 | 1.030 |
| **mechanical axis of lower limbs (mm)** | Right | 0.073 | 0.999 | 0.999 | 1.128 | 1.388 |
|  | Left | 0.213 | 0.999 | 0.999 | 1.014 | 1.312 |

**Table S6. Results of parameter calculation of**

**TKA lower limbs in the test set**

|  | | ***P* value** | **ICC** | **Pearson correlation** | **MAE** | **RMSE** |
| --- | --- | --- | --- | --- | --- | --- |
| **mLDFA ( °)** | Right | 0.194 | 0.969 | 0.968 | 0.594 | 0.839 |
|  | Left | 0.280 | 0.962 | 0.963 | 0.571 | 0.739 |
| **MPTA ( °)** | Right | 0.073 | 0.975 | 0.980 | 0.707 | 0.989 |
|  | Left | 0.084 | 0.989 | 0.990 | 0.418 | 0.577 |
| **LDTA ( °)** | Right | 0.662 | 0.914 | 0.925 | 1.940 | 2.388 |
|  | Left | 0.836 | 0.844 | 0.854 | 2.109 | 2.794 |
| **JLCA ( °)** | Right | 0.102 | 0.889 | 0.926 | 0.841 | 1.322 |
|  | Left | 0.065 | 0.941 | 0.949 | 0.531 | 0.729 |
| **mechanical axis of lower limbs (mm)** | Right | 0.938 | 0.999 | 0.999 | 1.052 | 1.395 |
|  | Left | 0.182 | 0.999 | 0.999 | 1.053 | 1.305 |

Fig S1. The measurement differences between AI predictions and ground truth in a patient with genu varum of the left knee. The figure shows the differences between AI predictions and ground truth for left lateral femoral condyle, medial femoral condyle, fossa intercondyle, lateral tibial condyle, medial tibial condyle and eminentia intercondyle in this patient.Red dot: ground truth; Blue dot: DCNN system


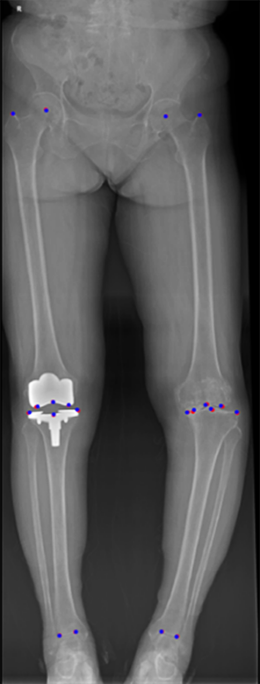


Fig S1
